# Augmented Renal Clearance, Muscle Catabolism and Urinary Nitrogen Loss: Implications for Nutritional Support in Critically Ill Trauma Patients

**DOI:** 10.3390/nu13103554

**Published:** 2021-10-11

**Authors:** Guilhem Dreydemy, Alexis Coussy, Alexandre Lannou, Laurent Petit, Matthieu Biais, Cédric Carrié

**Affiliations:** 1 Anesthesiology and Critical Care Department, CHU Pellegrin, 33000 Bordeaux, France; alexandre.lannou@chu-bordeaux.fr (A.L.); laurent.petit@chu-bordeaux.fr (L.P.); matthieu.biais@chu-bordeaux.fr (M.B.); cedric.carrie@chu-bordeaux.fr (C.C.); 2 Radiology Department, CHU Pellegrin, 33000 Bordeaux, France; alexis.coussy@chu-bordeaux.fr; 3 Health Department, University Bordeaux Segalen, 33000 Bordeaux, France

**Keywords:** augmented renal clearance, muscle wasting, nitrogen balance, protein intake, intensive care

## Abstract

The main objective of this pilot study was to determine the association between augmented renal clearance (ARC), urinary nitrogen loss and muscle wasting in critically ill trauma patients. We conducted a retrospective analysis of a local database in 162 critically ill trauma patients without chronic renal dysfunction. Nutritional-related parameters and 24 h urinary biochemical analyses were prospectively collected and averaged over the first ten days after admission. Augmented renal clearance was defined by a mean creatinine clearance (CL_CR_) > 130 mL/min/1.73 m^2^. The main outcome was the cumulated nitrogen balance at day 10. The secondary outcome was the variation of muscle psoas cross-sectional area (ΔCSA) calculated in the subgroup of patients who underwent at least two abdominal CT scans during the ICU length of stay. Overall, there was a significant correlation between mean CL_CR_ and mean urinary nitrogen loss (normalized coefficient: 0.47 ± 0.07, *p* < 0.0001). ARC was associated with a significantly higher urinary nitrogen loss (17 ± 5 vs. 14 ± 4 g/day, *p* < 0.0001) and a lower nitrogen balance (−6 ± 5 vs. −4 ± 5 g/day, *p* = 0.0002), without difference regarding the mean protein intake (0.7 ± 0.2 vs. 0.7 ± 0.3 g/kg/day, *p* = 0.260). In the subgroup of patients who underwent a second abdominal CT scan (N = 47), both ΔCSA and %ΔCSA were higher in ARC patients (−33 [−41; −25] vs. −15 [−29; −5] mm^2^/day, *p* = 0.010 and −3 [−3; −2] vs. −1 [−3; −1] %/day, *p* = 0.008). Critically ill trauma patients with ARC are thus characterized by a lower nitrogen balance and increased muscle loss over the 10 first days after ICU admission. The interest of an increased protein intake (>1.5 g/kg/day) in such patients remains a matter of controversy and must be confirmed by further randomized trials.

## 1. Introduction

Marked protein catabolism is considered of paramount importance during critical illness, especially in severe trauma patients [1]. Early targeted protein intake is thought to improve short-term outcome, reduce muscle wasting and hospital mortality [2,3]. Despite several limitations, determination of nitrogen balance is one of the most common methods to assess muscle catabolism and determine the targeted protein requirement [4,5].

On the other hand, critically ill trauma patients often experience augmented renal clearance (ARC), defined by an enhanced creatinine clearance (CL_CR_) exceeding 130 mL/min/1.73 m^2^ and responsible for an increased excretion of solutes and urinary eliminated medications [6]. The main physiological pathway leading to ARC involves the recruitment of renal functional reserve (RFR) through the inhibition of arteriolar vascular tone and increased renal blood [7,8]. Whether ARC is an adaptive response to acute aggression and/or a predisposing factor for further renal damage remains to be determined [9,10].

In this context, previous data demonstrated a close relationship between sarcopenia and renal hyperfiltration, mediated by several factors such as systemic inflammatory response syndrome (SIRS) and insulin resistance [11,12]. Based on this evidence, we hypothesized that ARC might be associated with increased urinary nitrogen loss, worsening the excessive protein breakdown resulting from enhanced muscle catabolism. The main objective of this pilot study was thus to determine the association between CL_CR_ and urinary nitrogen loss to better determine the targeted daily protein intake in critically ill trauma patients with or without ARC. The secondary objective was to explore the relationship between ARC and muscle wasting in critically ill trauma patients.

## 2. Materials and Methods

This pilot study is a retrospective analysis of our local database (declared to the French Data Protection Authority, declaration number 2166637v0) prospectively collected over a 20-month period (January 2019 to September 2020) in every critically ill trauma patient admitted in our 25-bed Surgical and Trauma Intensive Care Unit (ICU). Study participants had to have a length of stay ≥ 10 days, no history of chronic kidney disease and no need for renal replacement therapy.

Over the study period, management of nutrition therapy was consistent with the up-to-date recommendations [13]. Caloric and protein intakes (including calories from propofol or glucose infusion) were monitored daily in every patient. Enteral nutrition (EN) was introduced as soon as possible in the presence of a functional gastrointestinal tract after hemodynamic stabilization. The caloric delivery was progressively increased up to 80–100% of estimated needs, determined by adjusted weight-based predictive equations. A theoretical energy target ≥20–25 kcal/kg/day and a targeted protein administration ≥1–1.2 g/kg/day were considered adequate over the 10 first days after ICU admission. The caloric targets were updated by our dedicated dietician nutritionist, depending on previous nutritional status, occurrence of refeeding syndrome and other ICU-related medical conditions. Parenteral nutrition (PN) was implemented when enteral feeding was contraindicated if patients did not tolerate EN or when patients did not meet their nutritional targets within five to seven days.

For each patient, nutritional-related parameters and 24 h urinary biochemical analyzes were prospectively collected as part of the standard care and averaged over the study period [14]. A low-protein regimen was arbitrarily defined by a time-averaged protein intake ≤ 0.8 g/kg/day. Urinary creatinine clearance (CL_CR_) was calculated as follows: CL_CR_ = (24 h urinary volume x urinary creatinine)/(plasma creatinine), converted in mL/min and normalized to a body surface area of 1.73 m^2^ (Dubois formula). Augmented renal clearance was defined by a mean CL_CR_ > 130 mL/min/1.73 m^2^ over the study period [13]. The main outcome of the present study was the cumulated nitrogen balance at day 10, calculated using the daily protein intake and 24 h urine urea nitrogen (UUN) data (Table 1) [15,16]. Other covariates of interest were retrospectively collected in the computed medical record.

**Figure 1:**
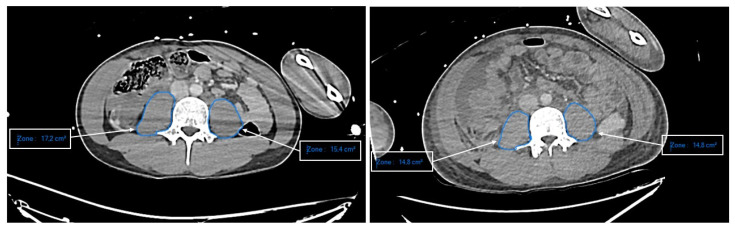
Example of muscle psoas cross-sectional area (CSA) measurement on the first (day 1) and the second (day 4) abdominal CT scan. Zone: Muscle psoas cross-sectional area calculated at the L4 level. Mean CSA at day 0: 1630 mm^2^; mean CSA at day 4: 1480 mm^2^. ΔCSA = 375 mm^2^/day; %ΔCSA = 5.8% per day.

Moreover, a retrospective image review was performed by a consultant radiologist in every patient with abdominal CT scans on hospital admission. Muscle psoas cross-sectional area (CSA) was calculated at the L4 level using the PACS measuring ruler (Figure 1) [17]. The right and left psoas muscle CSA were averaged to derive a mean value for each patient. Patients with psoas hematoma were excluded for analysis. For patients who had more than one abdominal CT scans during the ICU length of stay, the change (Δ) in CSA (mm^2^ per day) as well as %ΔCSA (% per day) were calculated [18].

Results are expressed as mean ± standard deviation or median (25% to 75% interquartile range) for continuous variables and as numbers (percentages) for categorical variables. The data distribution was analyzed by a Kolmogorov–Smirnov test. Continuous variables were compared using a Wilcoxon test for paired samples and categorical variables were compared using the chi-square test or Fisher’s exact test as appropriate. Longitudinal data were compared using a paired samples Wilcoxon test. For the main outcome of the study, multiple linear regression models were used to assess the correlation between time-averaged CL_CR_ and urinary nitrogen loss, with adjustment considered for age, total body weight, severity scores (SAPS 2, ISS) and mean protein intake. The theoretical daily protein intake was calculated for achievement of a positive nitrogen balance in patients with or without ARC according to predefined formulas (Table 1). A *p* value < 0.05 was considered statistically significant. Statistical analyses were performed using XLSTAT 2015 for Windows (Addinsoft, Paris, France).

## 3. Results

**Figure 2:**
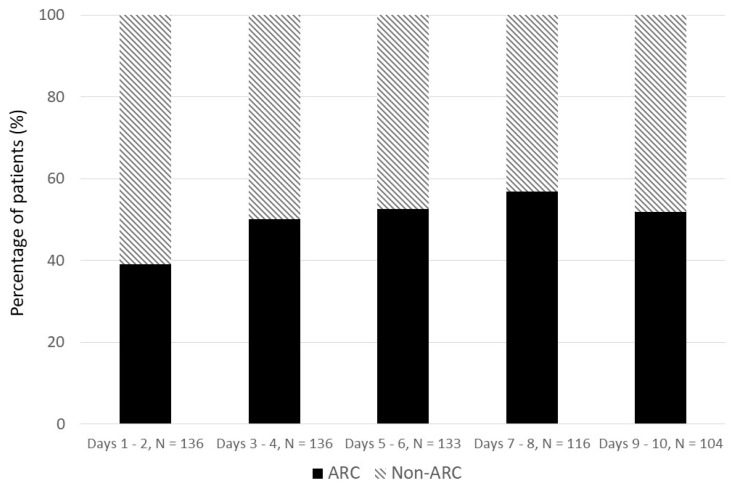
Incidence of ARC patients over the first ten days of ICU admission. ARC: mean CL_CR_ > 130 mL/min/1.73 m^2^ over the five study periods; N = number of urinary samples allowing CL_CR_ calculation over the five study periods.

Over the study period, 162 critically ill trauma patients were included and 75 (46%) demonstrated ARC over the first ten days after admission. The characteristics of the population, nutritional and biochemical parameters are listed in Table 2, Table 3 and Table 4. The final dataset consisted of 1125 individual urinary biochemical data. The incidence of ARC over the study period is depicted Figure 2. All patients had a complete collection of longitudinal nutritional data over the 10 first days after ICU admission.

There was a slight but significant correlation between mean CL_CR_ and mean urinary nitrogen loss over the study period (normalized coefficient: 0.47 ± 0.07, *p* < 0.0001). The only covariates associated with mean urinary nitrogen loss was TBW and ISS at admission (normalized coefficients: 0.42 ± 0.07, *p* < 0.0001 and 0.16 ± 0.07, *p* = 0.025), without statistical association with age, SAPS 2 or mean protein intake.

**Figure 3:**
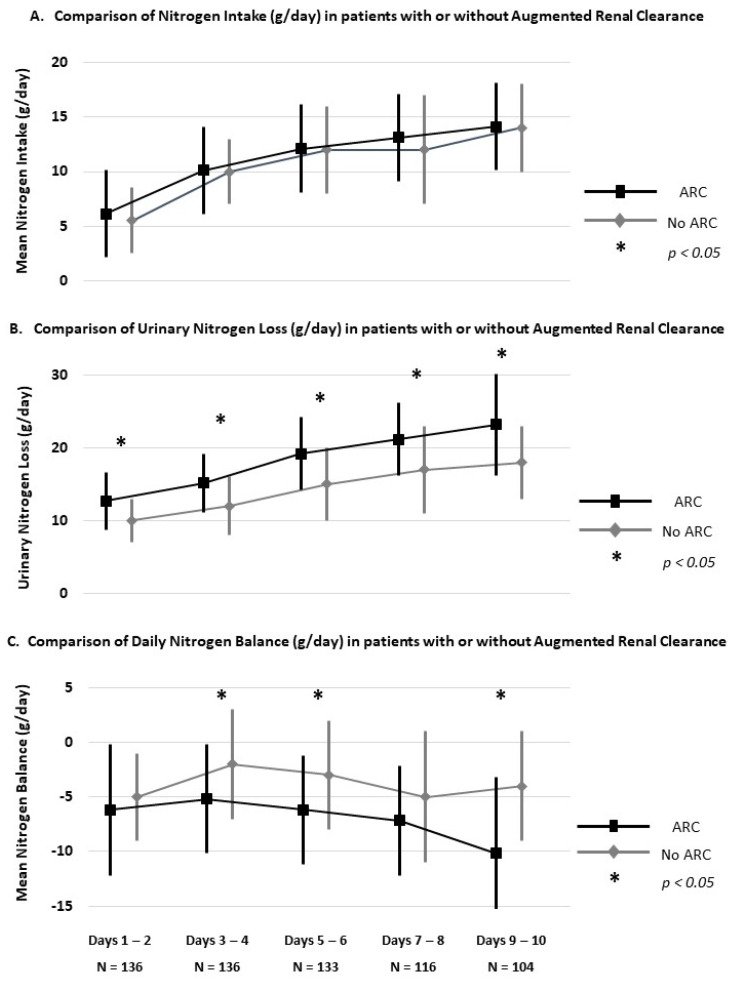
Distribution of nitrogen intake (**A**), urinary nitrogen loss (**B**) and nitrogen balance (**C**) compared in ARC vs. non-ARC patients over the study period. ARC: mean CL_CR_ > 130 mL/min/1.73 m^2^ over the five study periods; N = number of urinary samples allowing CL_CR_ calculation over the five study periods.

In this context, ARC was associated with a higher urinary nitrogen loss (17 ± 5 vs. 14 ± 4 g/day, *p* < 0.0001), leading to a lower nitrogen balance (−6 ± 5 vs. −4 ± 5 g/day, *p* = 0.0002), without a difference regarding the mean protein intake over the first 10 days after ICU admission (0.7 ± 0.2 vs. 0.7 ± 0.3 g/kg/day, *p* = 0.260). The longitudinal distribution of nitrogen intake, urinary nitrogen loss and nitrogen balance compared in ARC vs. non-ARC patients is depicted in Figure 3.

According to these results, patients with ARC require an increased targeted daily protein intake for achievement of a positive nitrogen balance compared to non-ARC patients (1.5 ± 0.3 vs. 1.2 ± 0.3 g/kg/day, *p* < 0.0001).

Over the study period, 129 abdominal CT scans upon hospital admission allowed adequate muscle psoas CSA. The muscle psoas CSA was similar between ARC vs. non-ARC patients at ICU admission (1004 ± 334 vs. 1104 ± 320 cm^2^, *p* = 0.135). Among these patients, 47 had a second abdominal CT allowing calculation of ΔCSA and %ΔCSA (within 7 [6; 11] days after ICU admission). In this subgroup of patients, both ΔCSA and %ΔCSA were higher in ARC patients (−33 [−41; −25] vs. −15 [−29; −5] cm^2^/day, *p* = 0.010 and −3 [−3; −2] vs. −1 [−3; −1] %/day, *p* = 0.008, respectively). Evolution of muscle psoas CSA over time between ARC vs. non-ARC patients is depicted in supplementary data (Appendix A).

Overall, 120 patients (74%) received a low protein intake over the first ten days after admission. Low protein intake was associated with a significantly lower nitrogen balance (−6 ± 4 vs. −2 ± 6, *p* < 0.0001), without statistical difference regarding the urinary nitrogen loss (16 ± 5 vs. 15 ± 4 g/day, *p* = 0.581). Patients with a mean protein administration ≥ 0.8 g/kg/day had significantly higher CR_CL_ than patients with low protein intake (138 ± 41 vs. 127 ± 36 mL/min/1.73 m^2^, *p* = 0.022). There was no statistical difference regarding ΔCSA and %ΔCSA in patients who received low vs. high protein intake (−20 [−33; −5] vs. −31 [−15; −41] cm^2^/day, *p* = 0.143 and −2 [−3; −1] vs. −3 [−3; −2] %/day, *p* = 0.078, respectively).

## 4. Discussion

To the best of our knowledge, this is one of the first studies exploring the relationship between augmented renal clearance, muscle catabolism and urinary nitrogen loss. In our cohort, critically ill trauma patients with ARC were characterized by a lower nitrogen balance and an increased muscle loss despite receiving a similar protein intake over the 10 first days after ICU admission. Patients with higher protein intakes were characterized by higher levels of CRCL, without statistical difference regarding ΔCSA and %ΔCSA compared to patients who received low protein intakes. These results are in accordance with a recent study suggesting a worsened nitrogen balance (−10.8 ± 13.0 vs. −6.2 ± 9.2 g/day, *p* = 0.004) in critically ill patients with ARC, although this study was impaired by the lack of muscle mass measurements and longitudinal data (only the first measurement being used for analysis) [5]. Of note, the authors also found a significant association between ARC and increased protein intake (adjusted OR: 2.06 [1.09, 3.91]). Taken together, these data may suggest that ARC is associated with an increased protein catabolism and muscle loss, although the retrospective design of these studies precludes any causal relationship. However, exploring the mechanisms of this association is of paramount importance in order to better determine the optimal protein intake in critically ill patients with ARC.

First, ARC and sarcopenia may share a common pathophysiological pathway, both mediated by the acute inflammatory response in patients with a greater functional reserve. Moreover, ARC may be a contributing factor of an enhanced urinary nitrogen loss, independently of age, total body weight, severity scores (SAPS 2, ISS) and mean protein intake. This result is supported by previous data reporting an increased urinary osmolality excretion, exceeding the tubular reabsorption capabilities in ARC patients [19]. In this regard, achievement of a positive nitrogen balance through a targeted protein intake has been extensively associated with improved outcomes during critical illness [20]. According to our results, patients with ARC would require an increased daily protein intake for achievement of a positive nitrogen balance compared to non-ARC patients (1.5 ± 0.3 vs. 1.2 ± 0.3 g/kg/day). Such a targeted protein intake should require high-protein-containing enteral nutrition, although the recent randomized controlled trials did not assess the effect on renal function, protein catabolism and muscle loss [21,22].

On the other hand, the excessive protein breakdown resulting from enhanced muscle catabolism might be a plausible physiologically pathway leading to ARC in critically ill patients. To support this hypothesis, the assessment of RFR in healthy subjects involves the stimulation of glomerular filtration rate (GFR) by an important protein intake either by oral diets or parenteral nutrition [23,24]. In critically ill patients, Doig et al. demonstrated that a daily intravenous infusion of 100 g of amino acids significantly improved GFR when compared to standard care in critically ill patients with renal dysfunction [25]. Hence, administration of a high protein diet could further increase renal hyperfiltration in trauma patients. In this context, nephrological data reported that a low-protein diet may exert reno-protection through the improvement of glomerular hyperfiltration due to the reduction of intra-glomerular pressure [26]. Moreover, former studies suggested that administration of high-protein diet was associated with a significant increase in protein oxidation and total energy expenditure, without an effect on muscle loss [27,28]. Whether the benefits of reno-protection outweigh the risk of protein–energy wasting and sarcopenia is not straightforward and deserves further studies.

Our study was impaired by several limitations. First, the retrospective design of this single-center study may lead to a selection bias and potential confounding factors, although limited by the longitudinal data and multiple linear regression models. Second, only few patients underwent a control CT scan, with variable completion times, thus limiting the conclusions about the muscle loss in this cohort of patients. Finally, we acknowledge that the total urinary nitrogen, the psoas CSA and the 24 h measured CL_CR_ are only surrogates of protein catabolism, muscle loss and glomerular filtration rate, with inherent limitations [15,29]. A prospective assessment of serum biomarkers of muscle wasting and glomerular filtration rate, such as myostatin and cystatin C, would be helpful for external validation of our hypothesis [30,31]. In this context, this study must be considered as a pilot study supporting the urgent need for more methodologically rigorous clinical trials examining the effects of high-protein nutrition supplements in critically ill trauma patients with or without ARC.

## 5. Conclusions

Critically ill trauma patients with ARC are characterized by a lower nitrogen balance and an increased muscle loss over the 10 first days after ICU admission. The interest of an increased protein intake (>1.5 g/kg/day) in such patients remains a matter of controversy and must be confirmed by further randomized trial.

## Figures and Tables

**Table 1:** Methods for calculation of urinary nitrogen loss and nitrogen balance [15,16].

**Urinary Nitrogen Loss (g/day)**	Urinary Urea [mmol/L] × 0.056/2.14 + 2 g/day (assumption of non-urea nitrogen)
**Total Nitrogen Loss (g/day)**	Urinary Nitrogen Loss + 2 g/day (assumption of extra-urinary Nitrogen Loss)
**Nitrogen Intake (g/day)**	Protein Intake [g/day]/6.25
**Nitrogen Balance (g/day)**	Nitrogen Intake—Total Nitrogen Loss

**Table 2:** Patient characteristics compared in ARC vs. non-ARC patients.

	ARC Population N = 75 (46)	Non-ARC Population N = 87 (54)	*p*
Demographic and Medical History			
Age (years)	34 [26; 50]	54 [35; 69]	<0.001
Male sex	64 (85)	63 (72)	0.046
TBW at ICU admission (kg)	74 [64; 84]	77 [69; 85]	0.105
BMI (kg/m^2^)	23 [21; 26]	26 [23; 29]	0.002
Charlson comorbidity index	0 [0; 1]	1 [0; 3]	<0.001
Poor nutritional status before admission *	7 (8)	6 (8)	0.991
Severity Scores			
Initial GCS	7 [4; 14]	11 [7; 15]	0.016
ISS	38 [25; 50]	43 [33; 51]	0.091
SAPS 2 at ICU admission	44 [35; 53]	44 [39; 58]	0.467
Traumatic Injuries (AIS > 3)			
Craniofacial trauma	56 (75)	63 (72)	0.746
Chest trauma	29 (39)	42 (48)	0.219
Abdominal trauma	20 (27)	29 (33)	0.357
Spine fracture	24 (32)	23 (26)	0.437
Patient Management and Complications			
Time under vasopressors	3 [1; 6]	4 [2; 6]	0.325
Time under sedation	2 [1; 6]	3 [1; 7]	0.184
Time under mechanical ventilation	14 [7; 19]	12 [7; 17]	0.509
Intracranial hypertension	26 (35)	23 (26)	0.284
ARDS	21 (28)	33 (38)	0.233
Need for antibiotics	66 (88)	75 (86)	0.886
Need for multiple surgeries	23 (31)	24 (28)	0.807
Need for multiple transfusion	22 (29)	36 (42)	0.114
Patient Outcome			
ICU mortality	1 (1)	9 (10)	0.017
ICU length of stay	22 [15; 33]	19 [13; 28]	0.173
TBW at ICU discharge	66 [56; 78]	67 [50; 80]	0.286
Weight Loss at ICU discharge, kg (%)	11 [4; 17]	10 [0; 22]	0.944

Results are expressed as numbers (percentage) and median [interquartile 25; 75]. TBW = total body weight; BMI = body mass index; AIS = Abbreviated Injury Scale; ARC = mean CLCR > 130 mL/min/1.73 m^2^ over the first ten days after ICU admission; GCS = Glasgow Coma Scale; ICU = intensive care unit; ISS = Injury Severity Score; SAPS 2 = Simplified Acute Physiology Score II. (*) Poor nutritional status is defined as follows: weight loss > 10% within 6 months, BMI < 20, ongoing oncological disease, chronic infectious disease or malabsorption syndrome. (≠) Results averaged over the first ten days.

**Table 3:** Nutritional parameters compared in ARC vs. non-ARC patients.

	ARC Population N = 75 (46)	Non-ARC Population N = 87 (54)	*p*
Use of enteral nutrition	74 (99)	84 (97)	0.387
Use of parenteral nutrition	27 (36)	49 (56)	0.010
Caloric Intake *, Kcals/kg/day (%)	20 [18; 24]	19 [17; 22]	0.019
Energy Target Achievement at Day 10 † (%)	81 [71; 96]	76 [66; 87]	0.019
Protein Intake *, g/kg/day	0.7 [0.6; 0.8]	0.7 [0.5; 0.8]	0.010
Protein Target Achievement at Day 10 † (%)	60 [53; 71]	55 [46; 64]	0.010

* Results averaged over the first ten days after ICU admission. (†) Mean percentage of energy target achievement and cumulative energy deficit calculated according to a theoretical energy target ≥25–30 kcal/kg/day and a targeted protein administration ≥1.2 g/kg/day over the 10 first days after ICU admission [12].

**Table 4:** Biological parameters and nitrogen balance compared in ARC vs. non-ARC patients.

	ARC Population N = 75 (46)	Non-ARC Population N = 87 (54)	*p*
Plasma Biological Analyses *			
Plasma Urea, mmol/L	5 [4; 6]	7 [5; 8]	<0.001
Plasma Creatinine, µmol/L	54 [47; 61]	63 [55; 74]	<0.001
Urea/Creatinine Ratio	97 [86; 117]	103 [84; 126]	0.485
Neutrophil-to-lymphocyte ratio	9 [6; 10]	7 [5; 9]	0.250
Urinary Biochemical Analyses *			
Urine Volume, L/day	2.5 [2.1; 3.0]	2.2 [1.8; 2.9]	0.036
Urinary Creatinine Excretion, mmol/L	7 [5; 8]	5 [4; 7]	0.027
Creatinine Clearance, mL/min/1.73 m^2^	158 [144; 170]	110 [87; 120]	<0.001
Urinary Urea Excretion, mmol/L	256 [177; 325]	228 [166; 281]	0.110
Urinary Nitrogen Loss, g/day	17 [14; 21]	14 [11; 17]	<0.001
Nitrogen Balance *			
Nitrogen Balance, g/day	−6 [−9; −3]	−4 [−6; −1]	<0.001
Cumulated Nitrogen Balance at Day 10, g	−56 [−92; −30]	−35 [−62; −13]	

* Results averaged over the first ten days after ICU admission. Results are expressed as numbers (percentage) and median [interquartile 25; 75]. ARC = mean CL_CR_ > 130 mL/min/1.73 m^2^ over the first ten days after ICU admission.

**Figure A1:**
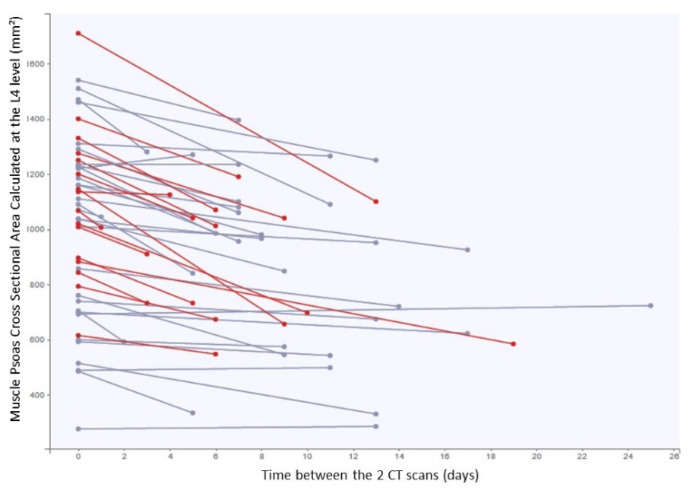
Evolution of muscle psoas CSA over time between ARC vs. non-ARC patients.

## Data Availability

The dataset used and analyzed for the current study is available from the corresponding author upon reasonable request.
